# Consumption of Tree Nuts as Snacks Reduces Metabolic Syndrome Risk in Young Adults: A Randomized Trial

**DOI:** 10.3390/nu15245051

**Published:** 2023-12-09

**Authors:** Kate Sumislawski, Annaliese Widmer, Robert R. Suro, Michelle E. Robles, Kate Lillegard, Dianna Olson, John R. Koethe, Heidi J. Silver

**Affiliations:** 1 Department of Medicine, Division of Gastroenterology, Hepatology, and Nutrition, Vanderbilt University Medical Center, Nashville, TN 37232, USA; 2 School of Medicine, Vanderbilt University, Nashville, TN 37232, USA; 3 Department of Medicine, Division of Infectious Diseases, Vanderbilt University Medical Center, Nashville, TN 37232, USA; john.r.koethe@vumc.org; 4 Department of Veterans Affairs, Tennessee Valley Healthcare System, Nashville, TN 37212, USA

**Keywords:** young adults, tree nuts, snacks, metabolic syndrome, insulin resistance

## Abstract

Metabolic syndrome (MetSx) and its chronic disease consequences are major public health concerns worldwide. Between-meal snacking may be a modifiable risk factor. We hypothesized that consuming tree nuts as snacks, versus typical carbohydrate snacks, would reduce risk for MetSx in young adults. A prospective, randomized, 16-week parallel-group diet intervention trial was conducted in 84 adults aged 22–36 with BMI 24.5 to 34.9 kg/m^2^ and ≥1 MetSx clinical risk factor. Tree nuts snacks (TNsnack) were matched to carbohydrate snacks (CHOsnack) for energy (kcal), protein, fiber, and sodium content as part of a 7-day eucaloric menu. Difference in change between groups was tested by analysis of covariance using general linear models. Multivariable linear regression modeling assessed main effects of TNsnack treatment and interactions between TNsnack and sex on MetSx score. Age, BMI, and year of study enrollment were included variables. There was a main effect of TNsnack on reducing waist circumference in females (mean difference: −2.20 ± 0.73 cm, *p* = 0.004) and a trend toward reduced visceral fat (−5.27 ± 13.05 cm^2^, *p* = 0.06). TNsnack decreased blood insulin levels in males (−1.14 ± 1.41 mIU/L, *p* = 0.05) and multivariable modeling showed a main effect of TNsnack on insulin. Main effects of TNsnack on triglycerides and TG/HDL ratio were observed (*p* = 0.04 for both) with TG/HDL ratio reduced ~11%. A main effect of TNsnack (*p* = 0.04) and an interaction effect between TNsnack and sex (*p* < 0.001) on total MetSx score yielded 67% reduced MetSx score in TNsnack females and 42% reduced MetSx score in TNsnack males. To our knowledge, this is the first randomized parallel-arm study to investigate cardiometabolic responses to TNsnacks versus typical CHOsnacks among young adults at risk of MetSx. Our study suggests daily tree nut consumption reduces MetSx risk by improving waist circumference, lipid biomarkers, and/or insulin sensitivity—without requiring caloric restriction.

## 1. Introduction

Metabolic syndrome (MetSx) and its associated cardiometabolic consequences have emerged as a major public health issue in most age groups. Of concern, the overall prevalence of MetSx has increased to 21.3% among American healthy young adults (aged 20–39 years) [1]. Concomitantly, data from the National Health and Nutrition Examination Surveys (NHANES) show a significant rise in average waist circumference, an indicator of abdominal adiposity [2] and a robust independent predictor for MetSx [3,4]. In several countries the trend in increased waist circumference and abdominal adiposity exceeds the relative increase observed in body mass index (BMI) [5], with the greatest escalation occurring in young adults [6]. In the state of high abdominal adiposity, hypertrophic dysfunctional subcutaneous fat is highly lipolytic, releasing free fatty acids to the viscera, the liver, and skeletal muscle. The accumulation of ectopic fat and its derivatives in organs and tissues impairs insulin action and signaling leading to a state of insulin resistance. Thus, insulin resistance is a key pathogenic link between excess adiposity and the physiological abnormalities that characterize MetSx: impaired glucose regulation, hypertension, hypertriglyceridemia, and low HDL-cholesterol levels [7].

While there is no one optimal diet for preventing MetSx, dietary macronutrient intake, particularly the amount of saturated fat (SFA) consumed, is a key factor in the development of obesity and insulin resistance. Indeed, replacing SFA with monounsaturated (MUFA) and/or polyunsaturated (PUFA) fats can improve insulin sensitivity as well as reduce blood pressure, serum triglycerides, and LDL-cholesterol levels [8,9]. A primary food source of MUFA and PUFA is tree nuts, of which the main fatty acids are oleic, linoleic, and α-linolenic acid. The fatty acid profile of tree nuts, along with other protective bioactive constituents, contributes to their beneficial effects on reducing individual MetSx risk factors, insulin resistance, overall MetSx risk, cardiovascular and coronary heart disease incidence, and all-cause mortality [10,11,12,13]. Although the high energy density of nuts has been a public health concern regarding potential weight gain, one meta-analysis showed a 3–5% reduced risk for developing overweight or obesity with each additional serving of tree nuts per day [14] and another meta-analysis showed no increase in overall adiposity quantified as body fat percentage [15]. Hence, the Dietary Guidelines for Americans recommendation for a healthy dietary pattern includes 4–6 ounces of nuts and seeds per week [16]—which may be consumed as between-meal snacks.

A survey of 27 countries conducted by the Harris Poll in 2022 shows 71% of consumers worldwide now consume snacks at least two times per day [17]. Nationally representative food intake data show a 28% increase in snacking in US adults from 1977 to 2006, with the greatest increase in daily energy intake per snack occurring in young adults [18]. Indeed, snacking contributes almost 25% of total daily caloric intake in US adults aged 20–39 years [19]. Evidence suggests that consuming tree nuts as between-meal snacks is a growing trend, particularly in North America, Europe, and Asia [20]. However, despite the evidence on improving health outcomes and national guidelines encouraging intake, nuts and seeds only comprise 5% of snack calories and only 35% of young adults consume tree nuts regularly [21]. In contrast, cookies, brownies, ice cream, cakes, pies, and candy are the main source of calories from snacks [22]. A study using NHANES data to model replacing the more typical high carbohydrate snacks with tree nuts indicates there would be a substantial improvement in diet quality with reduced intake of added sugars, saturated fats, and sodium, combined with increased intake of dietary fiber, magnesium, potassium, MUFA, PUFA, and omega-3 fatty acids [23].

This study was designed to compare the effects of consuming mixed tree nuts as snacks versus typical high carbohydrate food items as snacks without the confounding factor of caloric restriction and intentional weight loss. Given the relatively high rates of snacking among US young adults and the rising rates of overweight, obesity, and type 2 diabetes among this population subgroup [24], we focused on individuals aged 22–36 years who have at least one MetSx risk factor. We hypothesized that consuming tree nuts as snacks would reduce waist circumference, insulin resistance, and overall risk for metabolic syndrome.

## 2. Materials and Methods

### 2.1. Recruitment and Participants

**Figure 1:**
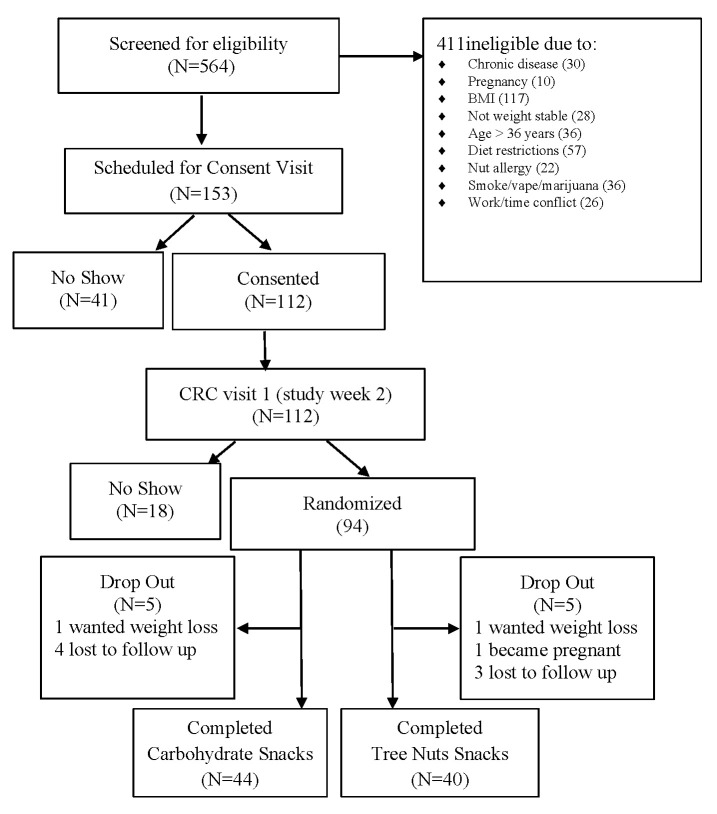
Schematic of study subject recruitment and retention.

Participants were recruited by Vanderbilt Diet, Body Composition, and Human Metabolism Core staff using the ResearchMatch.org registry, the VUMC research email list-serve, and flyers posted throughout the greater Nashville, TN metropolitan area. Eligibility criteria included adults age 22–36 years, BMI 24.5 to 34.9 kg/m^2^, weight stability within 3 pounds over the 3 months prior to enrollment, and having risk for metabolic syndrome (MetSx) based on cut points derived from adults aged 18–30 years in the CARDIA study (waist circumference ≥ 89 cm for males and ≥80 cm for females, serum triglyceride levels ≥ 128 mg/dL, high-density lipoprotein cholesterol (HDL) ≤ 40 mg/dL for males and <50 mg/dL for females, fasting serum glucose levels ≥ 100 mg/dL, and blood pressure values > 130/85 mm Hg) [25]. Potential participants were excluded if they had a tree nuts allergy, diagnosed chronic disease (type 1 or 2 diabetes, liver disease, kidney disease, lung disease, cardiovascular disease, cancer, polycystic ovary syndrome, irritable or inflammatory bowel syndrome, or celiac disease), or were prescribed medication for dyslipidemia or hyperglycemia, currently smoking, using illicit drugs, consuming excessive alcohol, pregnant or lactating (Figure 1).

### 2.2. Study Design

**Figure 2:**
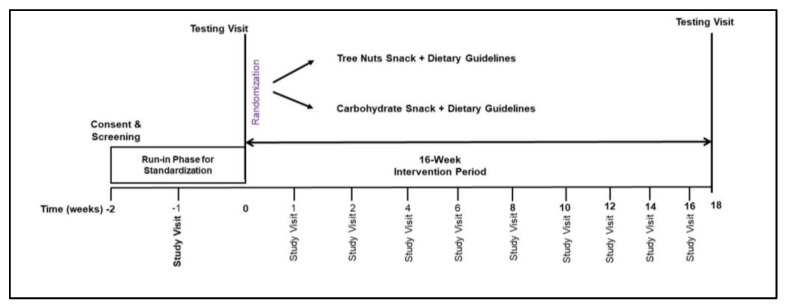
Randomized parallel treatment arms study design.

This study was a prospective, randomized, parallel-group diet intervention trial (Figure 2). Consenting of participants and screening laboratory values were performed at the Vanderbilt Center for Human Nutrition. Baseline data collection at the Vanderbilt Clinical Research Center included assessment of usual dietary intake, vital signs and anthropometrics, bloodwork, measurement of resting energy expenditure, imaging of the truncal region, and 7 days of physical activity monitoring. A two-week run-in period followed which included general nutrition counseling consistent with the 2020–2025 Dietary Guidelines [26]. Instruction to refrain from consuming all types of nuts and nut butters and a two-week supply of high carbohydrate snack food items were provided to all participants. Upon completion of the run-in period, participants were randomized to either the tree nuts snack group (TNsnack) or the high carbohydrate snack group (CHOsnack) for 16 weeks (Figure 2). Randomization was performed with unified reproducible methods using the R blockrand software package (https://www.R-project.org/ accessed on 15 January 2019) according to a permuted block randomization scheme with stratification by BMI group.

### 2.3. Diet and Snack Intervention

The intervention phase of the study was a 16-week period from study weeks 2 to 18. Menus based on the USDA Dietary Guidelines for Americans were designed using NDSR software (Nutrition Data System for Research, Univ. of Minn.) to create a 7-day cycle comprised of 3 meals and 2 snacks per day to be consumed between the hours of 6:00 a.m. to 6:00 p.m. Menus did not include any peanuts, tree nuts, or nut butter food items. Food portions were determined so that the energy content of the menu met each participant’s calorie goal for weight maintenance based on measured resting energy expenditure multiplied by a factor for physical activity energy expenditure. The macronutrient composition of the menus was within the Institute of Medicine acceptable distribution ranges of 25–35% fat, 45–55% carbohydrate, and 15–20% protein [27]. Snack calories in both the TNsnack and the CHOsnack condition were calculated to meet 15–20% of total daily energy needs. All tree nuts and carbohydrate snacks were portioned for individual snack consumption using food weight scales, packaged into plastic snack bags, and provided in shopping bags to participants every two weeks at their visit with the study dietitian. Tree nuts snacks were comprised of a 33.5 g mix of unsalted raw cashews, pistachios, hazelnuts, macadamia nuts, pecans, walnuts, and almonds. Carbohydrate snacks were matched to tree nuts snacks for energy (kcal), protein, fiber, and sodium content. They consisted of items such as unsalted pretzels, graham crackers, animal crackers, and nutrigrain/granola type bars. Assessments of dietary intakes were performed using the validated USDA multi-pass 24-h diet recall method with a standardized script [28]. Measuring utensils (plates, bowls, cups, spoons) of various sizes were used to prompt reliable estimation of portion sizes. Intakes were entered directly into NDSR software and analyzed for energy, macronutrient, and micronutrient content.

### 2.4. Anthropometrics and Computed Tomography Morphometrics

Height (±0.1 cm) was measured using a wall-mounted stadiometer, body weight (±0.1 kg) on a digital platform scale (Model 8437, Detecto, Webb City, MO, USA), and waist and hip circumferences (±0.1 cm) via flexible measuring tape with participants in light clothing without overgarments or shoes and pockets emptied. BMI, waist-to-hip, and waist-to-height ratios were calculated. Abdominal computed tomography (CT) scans were acquired without contrast on a GE Healthcare scanner and analyzed using the axial slice containing the inferior endplate of the 3rd lumbar vertebra. CT images were converted to Digital Imaging and Communications in Medicine (DICOM) format and segmentation of abdominal subcutaneous adipose tissue (SAT), visceral adipose tissue (VAT), and skeletal muscle (psoas, quadratus lumborum, erector spinae, lateral oblique, internal/external oblique, and rectus abdominus) was performed using an automated version of Slice-O-Matic software (version 4.3, Tomovision, Montreal, QC, Canada). Manual editing of tissue boundaries was performed by trained research technicians (coefficient of variation of 1.2%) to assure complete quantification of cross-sectional tissue depot areas. Radiodensities of SAT, VAT, and skeletal muscle were quantified based on established attenuation thresholds in Hounsfield Units as indicators of lipid accumulation in these tissues. VAT/SAT ratio was calculated as a metric that predicts cardiometabolic disease independent of VAT [29].

### 2.5. Resting Energy Expenditure

Resting energy expenditure (REE) was measured using a portable integrated metabolic cart system (ParvoMedics TrueOne 2400, Salt Lake City, UT, USA) calibrated to room air and a single gas tank prior to each use. Participants were instructed to fast from 9:00 pm the prior evening until 7:00 a.m. on the morning of testing, refrain from consuming alcohol and excess caffeine during the 24 h prior to arrival, and avoid non-routine physical activities. Thermoneutral conditions (ambient temperature, barometric pressure, and humidity) were achieved, and participants were habituated to the canopy hood for five minutes prior to testing. Data was collected for 20 min at steady state where average change in minute VO_2_ was ≤10% and average change in respiratory quotient (RQ) was ≤5%. REE in kilocalories was automatically calculated using the Weir equation [30] and substrate (fat and carbohydrate) oxidation rates were determined according to the method of Frayn with adjustment for 24-h urinary urea nitrogen output [31].

### 2.6. Physical Activity Monitoring

Physical activity levels were assessed using an ActiGraph wGT3X-BT accelerometer (ActiGraph, LLC Pensacola, FL, USA). Participants wore the SenseWear armband on their dominant wrist for one week at each study timepoint. Only days when the armband was worn for ≥12 h were used in analysis. Physical activity data was assessed using ActiLife software to provide time (minutes) spent sedentary and time in light, moderate, and vigorous intensity physical activities. In addition, total active time, percent of each day active, step counts per day, physical activity metabolic equivalents (METs), and energy (kcal) expended during physical activity were calculated.

### 2.7. Clinical Biomarkers

Blood pressure was measured in triplicate in a seated position after a 5-min rest period. Serum concentrations of high sensitivity C-reactive protein (hs-CRP), lipid profiles (triglycerides, total cholesterol, LDL-cholesterol, and HDL-cholesterol), glucose, and insulin were assayed at the Vanderbilt Department of Pathology Diagnostic Laboratory. The homeostasis model of insulin resistance (HOMA-IR) was calculated as [glucose (mg/dL) × insulin (uU/mL)/405].

### 2.8. Statistical Analysis

A priori power analysis was based on data from our prior diet intervention cohorts that showed a sample size of 50 completers per group would provide 80% probability to detect a mean difference of ≥2.54 cm in waist circumference, the primary outcome. Metabolic syndrome score (0–5) was calculated for each participant by assigning 1 point for each metabolic risk criteria met (waist circumference, glucose level, HDL-cholesterol level, triglyceride level, and blood pressure). Difference in changes between groups was tested by analysis of covariance (ANCOVA) using general linear models. Within group differences were assessed using the Wilcoxon test. Multivariable linear regression modeling was performed to assess the main effect of the tree nuts snack treatment and the interaction between treatment and sex on MetSx score. Each of the MetSx criteria and insulin level were also investigated as separate outcome variables. Age, BMI, and year of enrollment (the COVID-19 pandemic occurred during the study data collection period) were considered as between-subject factors but showed no main effect on any of the outcomes. Analyses were performed using SPSS^®^ Statistics (version 29.0.0, IBM, Armonk, NY, USA) with statistical significance set as *p* < 0.05.

## 3. Results

### 3.1. Baseline Characteristics of Study Sample

Of the 84 participants who completed the study, 65 (77.4%) self-identified as White and 19 (22.6%) as Black or other, and 48 (57.1%) were female. Participants had an average BMI of 28.4 ± 3.7 kg/m^2^ with 15.5% of participants meeting BMI criteria for normal weight, 58.3% for overweight, and 26.2% for obesity. All participants were normoglycemic (average fasting blood glucose: 84.5 ± 9.4 mg/dL) and normotensive (Table 1). Notably, the average baseline fasting insulin levels for all participants indicated a moderate state of hyperinsulinemia, with an average insulin level of 7.9 ± 7.2 mIU/L. The average waist circumference was 92.8 ± 10.5 cm in females and 102.2 ± 10.7 cm in males, with 65% of females and 50% of males meeting MetSx risk criteria for waist circumference, 14% of males and 8% of females meeting MetSx risk criteria for high triglyceride levels, and 65% of participants meeting MetSx risk criteria for low HDL-cholesterol levels. Overall, 64.3% of participants had at least one of the MetSx risk criteria, 25% had 2 risk criteria, and 10.7% had ≥3 risk criteria.

Baseline assessment of dietary intakes showed participants reported consuming 2253.2 ± 713.3 kcals/day, comprised of 49.2% carbohydrate, 34.2% fat, and 16.6% protein. Total sugars intake was ~93 g/day and added sugars 58.5 g/day, equating to ~15 teaspoons of added sugars per day. Regarding habitual tree nut consumption, 7.1% of participants reported daily consumption, 14.3% regular consumption (3–5 times per week), 40.5% had consumption of 1–2 times per week, 28.6% less than once per week, and 9.5% reported never consuming tree nuts. Physical activity monitoring revealed that participants were sedentary ~65% of the time. Of active time, 68% was spent performing light activities, 32% moderate activities, and <1% vigorous activities.

### 3.2. Effects of Snack Treatment on Dietary Intakes and Physical Activity

As planned with the study menus, no significant changes occurred over time in either group’s energy (calorie) intakes (Supplemental Table S1). There was a significant main effect of treatment on dietary fat intake with the TNsnack group consuming more dietary fat as a percentage of energy compared to the CHOsnack group (mean difference: 12.8 ± 3.2% kcals, *p* < 0.001). Both male and female TNsnack participants increased their total dietary fat intakes, by 13.03 ± 2.79% kcal in males and 8.55 ± 1.86% kcal in females (*p* < 0.001 for both). The increase in total dietary fat intakes in the TNsnack group derived from a significant increase in MUFA and PUFA, and thus, the ratio of unsaturated to saturated fats increased by 31% in females and males (*p* = 0.003 for both).

Concomitant with the increase in dietary MUFA and PUFA, the TNsnack group had significantly decreased intakes of carbohydrates as a percentage of energy, as well as decreased intakes of total starch, total sugars, and refined grains. The effect of consuming tree nuts as snacks on lowering total sugars intake was significant for females and males (mean difference between snack groups: −25.60 ± 16.86 g, *p* = 0.04). Further, the added sugars and sucrose intakes of male TNsnack participants were significantly reduced compared to male CHOsnack participants (mean difference in added sugars: −14.74 ± 16.13 g, *p* = 0.05; mean difference in sucrose −16.18 ± 11.26, *p* = 0.01). No significant change occurred in sodium intakes in either group.

Overall, there were no significant changes between groups in the amount of time spent in physical activities, intensity of physical activities performed, or physical activity energy expenditure. Males in the TNsnack group increased time spent in moderate intensity activities (19.17 ± 25.21 min, *p* = 0.04). However, they also decreased the total number of steps taken per day (−1262.46 ± 1640.40 steps, *p* = 0.04). Females in the TNsnack group decreased their total physically active time (−56.68 ± 77.69 min, *p* = 0.04) and increased their sedentary time (53.13 ± 54.77 min, *p* = 0.002). No significant changes were observed in the CHOsnack group for physical activity factors (Table 2).

### 3.3. Effects of Snack Treatment on Weight, Waist Circumference, Body Composition, and Energy Expenditure

TNsnack female participants experienced a non-significant decrease in weight and BMI (−0.84 ± 1.55 kg, *p* = 0.08 and −0.39 ± 0.94 units, *p* = 0.10, respectively) in parallel with CHOsnack female participants having a significant increase in weight (0.95 ± 1.52 kg, *p* = 0.006) and BMI (0.44 ± 0.54 units, *p* = 0.005). There was a significant main effect of TNsnack on waist circumference among females (mean difference: −2.20 ± 0.73 cm, *p* = 0.004), which resulted from a decrease of −1.59 ± 2.31 cm (*p* = 0.003) in waist circumference. Although not statistically significant, females in the TNsnack group had decreased hip circumference while those in the CHOsnack group had increased hip circumference. Likewise, the decrease in VAT area in TNsnack females (−5.27 ± 13.05 cm^2^, *p* = 0.06) occurred concomitantly with increased VAT in CHOsnack females (4.49 ± 13.08 cm^2^, *p* = 0.10). There were no significant changes in anthropometric measures among males in either snack group.

There were no significant changes in REE within or between groups (Table 3). In the TNsnack group, carbohydrate oxidation decreased (males: −6.19 ± 6.96% REE kcal; females: −6.46 ± 6.67% REE kcal, *p* < 0.001 for both) while fat and protein oxidation increased. Conversely, in the CHOsnack group, carbohydrate oxidation increased (males: 4.77 ± 2.63% REE kcal; females: 3.83 ± 3.39% REE kcal, *p* < 0.001 for both) along with decreased fat oxidation.

### 3.4. Effects of Snack Treatment on Clinical Biomarkers and Metabolic Syndrome Risk Score

Serum CRP levels increased in the female CHOsnack group by 0.57 ± 1.17 mg/dL (*p* = 0.03), which yielded a significant mean difference between groups of 1.00 ± 0.45 mg/dL (*p* = 0.04). Although the direction of change also differed between snack groups in males, the difference between groups was not significant in males. Systolic blood pressure increased in CHOsnack males (3.15 ± 7.67 mmHg, *p* = 0.05), which resulted in a significant difference in the change between snack groups in males (4.92 ± 2.85 mmHg, *p* = 0.04). 

Likewise, the direction of the change in fasting blood glucose levels differed between snack groups in males, resulting in a mean difference between snack groups of 5.23 ± 4.66 mg/dL (*p* = 0.03). A similar tendency was observed in blood insulin levels in male participants with the TNsnack group having a reduction of 1.14 ± 1.41 mIU/L (*p* = 0.05) and a non-significant decrease in TNsnack females (−1.52 ± 1.78, *p* = 0.13). However, multivariable modeling accounting for age, BMI, and year of data collection showed a main effect of TNsnack on insulin levels (Table 3).

There were no main effects of tree nuts snacks on cholesterol levels and the reductions observed in total, LDL-cholesterol, and HDL-cholesterol were not statistically significant in either snack group. Multivariable modeling showed a main effect of tree nuts snacks on triglycerides and the TG/HDL ratio (*p* = 0.04 for both, Table 3), with TG/HDL ratio reduced ~11% in TNsnack participants. MetSx score reduced significantly in the TNsnack group so that there was a main effect of tree nuts snacks (*p* = 0.04) and an interaction effect between tree nuts snacks and sex (*p* < 0.001), yielding a 67% reduction in MetSx score in TNsnack females and a 42% reduction in MetSx score in TNsnack males.

## 4. Discussion

This randomized parallel design eucaloric diet intervention study had several key findings. First, daily consumption of tree nuts as between-meal snacks significantly reduced total metabolic syndrome risk score in young adults, most of whom met BMI criteria for overweight or obesity and had at least one METSx risk factor at baseline. Although this is the first trial comparing consumption of tree nuts as snacks versus typical carbohydrate snacks in young adults, the reduction in MetSx score is consistent with NHANES data showing lower prevalence of MetSx in tree nut consumers [32], findings from the PREDIMED trial showing decreased risk for MetSx in individuals with high cardiovascular disease risk [33], and the Tehran Lipid and Glucose study which showed reduced incidence of MetSx with tree nut consumption of five or more servings per week [34].

Evidence from nutrient analysis studies suggests that tree nuts offer substantial protective cardiometabolic health benefits due to their compositional characteristics [35]. The fatty acid profile of tree nuts is mainly comprised of the monounsaturated fat oleic acid and the polyunsaturated fat linoleic acid. Tree nuts are also rich sources of protein, fiber, vitamins E and K, minerals, carotenoids, tocopherols, polyphenols, and phytosterols. When consumed as between-meal snacks, these components may displace the intake of nutrients often associated with increased cardiometabolic disease risk, i.e., saturated fats and sugars, and thus, improve overall diet quality [23]. In the present study, we observed a 31% increase in the ratio of unsaturated to saturated fat intake along with a 29% decrease in total sugars intake in the TNsnack group. In addition, the TNsnack group had significant increases in their vitamin E, folate, vitamin B6, calcium, magnesium, potassium, and zinc intakes.

Despite being a plant based food, the high energy density (kcal/g) of tree nuts, due to dietary fat content, has raised concern for a potential weight gaining effect—especially in the current environment where young adults (20–39 years) are the age group at greatest risk for developing overweight and obesity [36]. However, a meta-analysis that included randomized feeding trials and prospective cohort studies showed a 4% reduced relative risk for overweight and obesity with each additional weekly serving of tree nuts [14]. In contrast to most trials that incorporate caloric restriction, the present study was designed to be eucaloric to enable investigation of an independent effect of tree nuts consumption on body weight. We observed no change in energy intake or body weight in the TNsnack group. Notably, feeding trials have shown that consumption of tree nuts promotes satiety, reducing hunger and desire to eat while increasing the sensation of fullness, particularly when consumed as between-meal snacks [37,38]. Whether the beneficial effects of tree nuts consumption is influenced by the specific timing and duration of food intake remains to be explored [39].

Along with the rise in the prevalence of overweight and obesity in young adults, data from NHANES shows the largest increase in waist circumference, a proxy for abdominal adiposity, has been occurring in the young adult age group [40]. Indeed, the incidence of excess abdominal adiposity has more than doubled in this age group over the past 3 decades. At study completion, the TNsnack group had significantly reduced waist circumference, which differed from the change in the CHOsnack group, and occurred primarily in female TNsnack participants. At any given waist circumference, females are likely to have more total abdominal adipose tissue, with more abdominal SAT but less VAT than males [41]. Although different in biochemical and molecular characteristics, dysregulation of both SAT and VAT contribute to the metabolic syndrome, correlating with disruption of free fatty acid metabolism, ectopic fat deposition in organs and skeletal muscle, increased inflammation, and insulin resistance [42]. Although not statistically significant, we observed a trend towards reduced VAT in the TNsnack female participants. While investigation of the effects of tree nuts consumption on adipose tissue depots in human is lacking, studies in animal and rodent models indicate positive effects on abdominal fat deposition [43,44] and adipocyte differentiation [45]. Despite that we detected no effects of the TNsnack on the physical activity parameters measured, other evidence in young adults suggests that other components of physical fitness such as muscle strength and cardiorespiratory fitness may mediate the relationship between tree nuts intake and body composition [46].

Another mechanism by which increased tree nut consumption could exert beneficial metabolic health effects and reduce MetSx risk is by altering lipid metabolism and reducing dyslipidemia. A systematic review and meta-analysis of 10 randomized controlled trials in persons with overweight or obesity showed significant reductions in serum LDL-cholesterol and triglycerides (TG) levels [47]. While our data showed no significant effects of consuming tree nuts snacks on LDL or HDL-cholesterol, multivariable regression modeling showed a significant main effect of tree nuts snacks on TG as well as the TG/HDL ratio, a more robust biomarker for identifying MetSx than other lipid ratios [48] and a reliable biomarker for identifying insulin resistance [49]. In addition to potential effects on lipids and lipoproteins, the high unsaturated fatty acid content of tree nuts may affect fatty acid β-oxidation. Investigations in rodent models and humans have shown more rapid oxidation of unsaturated versus saturated fats [50,51,52]. One study showed that high unsaturated fat intake can improve fatty acid oxidation rate back to normal levels in adults with overweight and obesity [53]. Increased fat oxidation in TNsnack participants occurred in parallel with decreased fat oxidation in CHOsnack participants. The finding of higher fat oxidation with tree nuts snack consumption is consistent with TNsnack participants having no increase in body weight or body fat.

A meta-analysis of the effects of tree nuts on glycemic indexes showed that tree nut consumption yields a modest reduction in fasting insulin levels and insulin resistance (based on HOMA-IR score), but does not change fasting glucose levels [54]. Similarly, we observed no significant changes in glucose levels, but a significant main effect of tree nuts snack consumption on insulin levels, particularly in male participants. However, no significant effect was detected for overall HOMA-IR score. While insulin levels are not part of the cluster of risk factors used to identify MetSx, insulin resistance is considered a strong underlying contributor and impaired insulin action key to the development of glucose and lipid dysregulation, promoted by excessive accumulation of abdominal adipose tissue. Notably, high saturated fat intake is associated with inhibition of insulin action in insulin responsive tissues whereas high unsaturated fat intake increases insulin sensitivity.

Strengths of this study include the randomized parallel arm design and the application of a pragmatic real-world dietary snack intervention, especially at a time when the portion size and frequency of snack consumption contributes to almost one-fourth of daily caloric intake in young and middle-aged adults. Additionally, effects of tree nuts as snacks were investigated without caloric restriction, which would mask any beneficial direct effects of tree nuts. Thirdly, the intervention and support participants received from registered dietitians was similar for TNsnack and CHOsnack groups. A limitation of the study is the relatively small sample size per group which makes data analysis statistically underpowered to detect differences between groups for variables that have large variability. Thus, several data trends observed require further investigation with larger samples. Secondly, the intervention period of 16 weeks is likely too short a duration to detect changes in CT-determined morphometrics. Finally, it is well-recognized that diet assessment via self-reporting, despite using validated methodology, is susceptible to under or over-reporting bias.

## 5. Conclusions

While there are several studies that investigate the effects of tree nut consumption on energy metabolism and abdominal obesity, to our knowledge this is the first randomized parallel-arm study to investigate the cardiometabolic responses to tree nut snack consumption in comparison to traditional carbohydrate-rich snacks among young adults at risk of metabolic syndrome. Notably, TNsnack consumption improved diet quality by increasing the ratio of unsaturated to saturated fat intake and reducing total sugars intake. Overall, our study suggests that daily tree nut consumption reduces metabolic syndrome risk without the requirement of caloric restriction, potentially by improving waist circumference, lipid biomarkers, and insulin sensitivity. Given the widespread prevalence of metabolic syndrome and its harmful potential for the development of diabetes and cardiovascular diseases, it is imperative that practical and feasible therapeutic strategies be adopted. Future investigation could be conducted in subgroups of the young adult population such as athletes as well as populations with chronic disease states including prediabetes, type 2 diabetes, and cardiovascular diseases.

## Figures and Tables

**Table 1:** (a). Changes in Anthropometrics and Clinical Biomarkers in Males (n = 36). (b). Changes in Anthropometrics and Clinical Biomarkers in Females (n = 48).

a. Males								
	Tree Nuts Group			CHO Group			Treatment Effect	
	Baseline	Change	*p*-Value	Baseline	Change	*p*-Value	Mean Difference	*p*-Value
Height (cm)	179.52 ± 8.55	n/a	n/a	177.88 ± 6.46	n/a	n/a	1.64 ± 2.50	0.52
Weight (kg)	93.66 ± 12.63	0.29 ± 4.32	0.80	92.87 ± 14.53	0.57 ± 2.39	0.30	0.28 ± 1.20	0.80
Body Mass Index (kg/m^2^)	29.03 ± 3.26	0.13 ± 1.23	0.67	29.28 ± 3.75	0.18 ± 0.78	0.32	0.05 ± 0.34	0.89
Waist Circumference (cm)	101.95 ± 9.37	−0.78 ± 1.11	0.15	102.33 ± 12.00	−0.14 ± 2.17	0.77	0.64 ± 0.91	0.47
Hip Circumference (cm)	109.06 ± 6.33	−0.37 ± 4.03	0.72	110.41 ± 6.56	−0.26 ± 3.42	0.74	0.11 ± 1.24	0.93
Waist:Hip (ratio)	0.94 ± 0.08	−0.01 ± 0.06	0.44	0.92 ± 0.06	−0.01 ± 0.02	0.16	0.00 ± 0.02	0.77
Waist:Height (ratio)	0.57 ± 0.05	−0.01 ± 0.03	0.31	0.58 ± 0.06	−0.00 ± 0.02	0.79	0.00 ± 0.01	0.75
Systolic Blood Pressure (mm Hg)	124.83 ± 9.69	−1.78 ± 9.46	0.47	119.90 ± 11.02	3.15 ± 7.67	0.05	4.92 ± 2.85	0.04
Diastolic Blood Pressure (mm Hg)	75.14 ± 8.07	−0.29 ± 7.55	0.88	75.15 ± 7.41	0.25 ± 7.95	0.89	0.44 ± 2.58	0.68
Physical Activity Level	11.59 ± 7.67	−0.73 ± 3.85	0.46	8.20 ± 3.64	−1.11 ± 6.31	0.44	0.44 ± 1.80	0.61
Glucose (mg/dL)	88.88 ± 12.03	−1.38 ± 1.19	0.22	83.20 ± 8.83	3.00 ± 3.53	0.10	5.23 ± 4.66	0.03
Insulin (mIU/L)	8.06 ± 7.63	−1.14 ± 1.41	0.05	6.45 ± 4.04	1.08 ± 4.09	0.25	2.22 ± 1.60	0.09
HOMA-IR (score)	1.86 ± 1.97	−0.25 ± 1.57	0.54	1.34 ± 0.89	0.28 ± 0.99	0.22	0.54 ± 0.43	0.11
C Reactive Protein (mg/dL)	2.01 ± 3.99	−0.49 ± 4.34	0.66	2.39 ± 3.12	0.71 ± 2.24	0.17	1.20 ± 1.12	0.29
Total Cholesterol (mg/dL)	165.44 ± 31.92	−1.89 ± 22.64	0.75	174.05 ± 33.82	−2.00 ± 30.51	0.77	0.13 ± 9.16	0.98
HDL-Cholesterol (mg/dL)	48.44 ± 16.64	−2.50 ± 9.17	0.20	46.65 ± 10.53	−1.30 ± 6.78	0.40	1.10 ± 2.66	0.69
LDL-Cholesterol (mg/dL)	98.38 ± 25.17	−2.31 ± 19.01	0.36	107.75 ± 32.16	4.05 ± 25.68	0.49	6.36 ± 7.45	0.20
Triglycerides (mg/dL)	92.94 ± 58.15	−8.81 ± 22.78	0.14	98.45 ± 46.20	6.25 ± 50.13	0.58	15.06 ± 13.56	0.15
TG:HDL (ratio)	2.29 ± 1.96	−0.25 ± 1.17	0.19	2.32 ± 1.38	4.09 ± 6.68	0.58	4.06 ± 4.92	0.02
Metabolic Syndrome (score)	1.94 ± 1.23	−0.81 ± 0.54	0.003	1.70 ± 0.89	0.05 ± 0.60	0.27	0.86 ± 0.19	< 0.001
**b. Females**								
	**Tree Nuts Group**			**CHO Group**			**Treatment Effect**	
	**Baseline**	**Change**	***p*-value**	**Baseline**	**Change**	***p*-value**	**Mean Difference**	***p*-value**
Height (cm)	165.83 ± 7.75	n/a	n/a	165.53 ± 8.02	n/a	n/a	0.29 ± 2.28	0.90
Weight (kg)	77.91 ± 12.07	−0.84 ± 1.55	0.08	74.67 ± 12.78	0.95 ± 1.52	0.006	1.72 ± 0.61	0.08
Body Mass Index (kg/m2)	28.31 ± 3.82	−0.39 ± 0.94	0.10	27.17 ± 3.59	0.44 ± 0.54	0.005	0.05 ± 0.22	0.33
Waist Circumference (cm)	94.36 ± 9.94	−1.59 ± 2.31	0.003	90.44 ± 10.77	0.60 ± 2.74	0.29	2.20 ± 0.73	0.004
Hip Circumference (cm)	110.08 ± 8.25	−0.61 ± 3.23	0.37	107.30 ± 8.29	0.95 ± 3.25	0.17	1.04 ± 0.93	0.05
Waist:Hip (ratio)	0.86 ± 0.06	−0.01 ± 0.03	0.32	0.84 ± 0.06	−0.00 ± 0.04	0.95	0.01 ± 0.00	0.57
Waist:Height (ratio)	0.57 ± 0.06	−0.01 ± 0.02	0.30	0.55 ± 0.06	−0.00 ± 0.02	0.98	0.01 ± 0.01	0.44
Systolic Blood Pressure (mm Hg)	112.70 ± 9.87	−0.23 ± 10.06	0.92	111.92 ± 8.23	−0.22 ± 8.51	0.86	0.09 ± 2.81	0.97
Diastolic Blood Pressure (mm Hg)	73.52 ± 9.89	−1.73 ± 8.26	0.34	72.58 ± 7.37	−0.59 ± 6.41	0.67	1.14 ± 2.23	0.61
Physical Activity Level	9.28 ± 3.94	−0.84 ± 1.05	0.43	8.56 ± 5.50	−0.62 ± 1.56	0.82	0.22 ± 1.40	0.66
Glucose (mg/dL)	84.58 ± 9.04	−1.08 ± 1.65	0.49	82.71 ± 7.88	0.82 ± 1.92	0.33	1.29 ± 2.68	0.63
Insulin (mIU/L)	10.15 ± 11.18	−1.52 ± 1.78	0.13	7.13 ± 2.95	0.65 ± 1.92	0.13	1.87 ± 1.67	0.07
HOMA-IR (score)	2.27 ± 3.04	−0.60 ± 0.42	0.15	1.49 ± 0.70	0.13 ± 0.56	0.27	0.77 ± 0.44	0.19
C Reactive Protein (mg/dL)	3.08 ± 2.78	−0.52 ± 1.89	0.19	1.39 ± 1.65	0.57 ± 1.17	0.03	1.00 ± 0.45	0.04
Total Cholesterol (mg/dL)	187.63 ± 39.09	−7.04 ± 28.31	0.24	175.83 ± 35.84	−6.17 ± 31.39	0.33	0.95 ± 8.45	0.92
HDL-Cholesterol (mg/dL)	54.33 ± 14.31	−2.46 ± 9.64	0.22	57.58 ± 13.71	−1.04 ± 7.63	0.52	1.41 ± 2.53	0.58
LDL-Cholesterol (mg/dL)	115.29 ± 34.22	−6.96 ± 31.38	0.29	102.25 ± 31.40	−2.43 ± 23.91	0.63	4.52 ± 8.16	0.18
Triglycerides (mg/dL)	103.67 ± 59.56	−6.54 ± 38.83	0.42	80.17 ± 32.38	−13.54 ± 25.93	0.02	7.16 ± 9.67	0.01
TG:HDL (ratio)	2.11 ± 1.51	−0.24 ± 0.49	0.02	1.50 ± 0.72	0.07 ± 0.93	0.03	0.36 ± 0.25	0.05
Metabolic Syndrome (score)	1.58 ± 0.83	−1.06 ± 0.19	0.005	1.33 ± 0.38	0.00 ± 0.10	<0.001	1.05 ± 0.13	0.001

**Table 2:** (a). Changes in Physical Activity, Energy Expenditure, and Body Composition Factors in Males (n = 36). (b). Changes in Physical Activity, Energy Expenditure, and Body Composition Factors in Females (n = 48).

a. Males								
	Tree Nuts Group			CHO Group			Treatment Effect	
	Baseline	Change	*p*-Value	Baseline	Change	*p*-Value	Mean Difference	*p*-Value
Sedentary time (min)	793.79 ± 124.05	17.44 ± 54.17	0.71	748.86 ± 134.89	3.92 ± 13.83	0.91	14.44 ± 18.64	0.68
Light activity time (min)	320.26 ± 83.16	−41.75 ± 81.68	0.14	304.65 ± 71.62	−20.82 ± 77.23	0.31	20.94 ± 29.63	0.31
Moderate activity time (min)	163.36 ± 78.73	19.17 ± 25.21	0.04	152.20 ± 43.04	16.59 ± 32.16	0.37	2.58 ± 19.56	0.86
Vigorous activity time (min)	0.00 ± 0.00	0.00 ± 0.00	0.99	0.00 ± 0.00	0.00 ± 0.00	0.99	0.00 ± 0.00	0.99
Total Active Time (min)	483.62 ± 135.07	−29.92 ± 41.79	0.11	455.95 ± 101.51	−4.42 ± 11.65	0.46	24.49 ± 37.35	0.25
Percent of Day Active (%)	33.59 ± 9.38	−0.07 ± 0.12	0.57	31.66 ± 7.05	−0.02 ± 0.09	0.75	0.05 ± 0.04	0.09
Steps per Day (count)	10,827.61 ± 3391.96	−1262.46 ± 1640.40	0.04	9643.29 ± 2543.50	−729.61 ± 3371.44	0.42	−532.85 ± 1013.34	0.60
Physical Activity METs	1.503 ± 0.253	−0.06 ± 0.09	0.08	1.447 ± 0.128	−0.03 ± 0.18	0.21	0.02 ± 0.06	0.72
Physical Activity Energy Expenditure (kcal)	1570.55 ± 918.31	−100.50 ± 151.65	0.07	1273.14 ± 433.67	−38.86 ± 49.74	0.77	61.63 ± 75.40	0.36
Resting Energy Expenditure (kcal)	1921.0 ± 272.02	−0.13 ± 262.00	0.85	1899.79 ± 248.80	50.94 ± 196.14	0.27	64.01 ± 77.51	0.42
Respiratory Quotient	0.83 ± 0.06	0.01 ± 0.09	0.71	0.81 ± 0.04	0.02 ± 0.06	0.29	0.02 ± 0.03	0.35
Carbohydrate Oxidation (% REE kcal)	40.13 ± 17.30	−6.19 ± 6.96	<0.001	30.26 ± 14.13	4.77 ± 2.63	<0.001	0.07 ± 4.72	0.98
Fat Oxidation (% REE kcal)	43.67 ± 17.09	4.35 ± 4.63	<0.001	52.82 ± 14.68	−8.48 ± 8.71	<0.001	26.13 ± 15.70	0.34
Protein Oxidation (% REE kcal)	16.51 ± 7.46	2.30 ± 2.64	<0.001	17.34 ± 7.91	4.11 ± 6.25	<0.001	4.19 ± 2.70	0.13
Visceral Adipose Tissue Area (cm)	109.98 ± 65.63	−4.12 ± 4.96	0.12	111.19 ± 60.65	−2.58 ± 28.26	0.69	1.58 ± 10.04	0.67
Subcutaneous Adipose Tissue Area (cm)	242.51 ± 101.09	−12.15 ± 14.98	0.29	274.40 ± 141.74	−10.08 ± 82.66	0.59	2.23 ± 2.44	0.53
VAT:SAT (ratio)	0.45 ± 0.22	−0.02 ± 0.11	0.43	0.46 ± 0.22	0.01 ± 0.12	0.51	0.03 ± 0.09	0.43
**b. Females**								
	**Tree Nuts Group**			**CHO Group**			**Treatment Effect**	
	**Baseline**	**Change**	***p*-value**	**Baseline**	**Change**	***p*-value**	**Mean Difference**	***p*-value**
Sedentary time (min)	703.36 ± 113.03	53.13 ± 54.77	0.002	802.22 ± 163.23	−10.14 ± 99.66	0.69	49.29 ± 36.74	0.19
Light activity time (min)	367.76 ± 112.46	−83.74 ± 110.20	0.01	336.20 ± 97.40	−35.96 ± 103.85	0.19	47.78 ± 38.44	0.23
Moderate activity time (min)	169.99 ± 61.44	31.95 ± 43.85	0.04	152.53 ± 59.55	−12.19 ± 41.41	0.26	43.24 ± 85.47	0.42
Vigorous activity time (min)	0.00 ± 0.00	0.00 ± 0.00	0.99	0.00 ± 0.00	0.00 ± 0.00	0.99	0.00 ± 0.00	0.99
Total Active Time (min)	538.44 ± 113.71	−56.68 ± 77.69	0.04	488.74 ± 138.19	−46.55 ± 117.32	0.13	10.13 ± 19.42	0.75
Percent of Day Active (%)	37.39 ± 7.90	−0.06 ± 0.08	0.62	33.94 ± 9.60	-0.04 ± 0.09	0.44	0.02 ± 0.02	0.35
Steps per Day (count)	10,468.64 ± 2599.30	−825.21 ± 2107.47	0.15	10,126.04 ± 2727.41	−780.32 ± 2222.45	0.18	44.88 ± 779.07	0.95
Physical Activity METs	1.468 ± 0.151	−0.06 ± 0.08	0.03	1.396 ± 0.145	−0.01 ± 0.09	0.90	0.05 ± 0.03	0.11
Physical Activity Energy Expenditure (kcal)	1236.56 ± 366.22	−192.12 ± 248.54	0.11	1055.16 ± 342.82	−8.92 ± 284.69	0.92	181.85 ± 96.26	0.05
Resting Energy Expenditure (kcal)	1508.92 ± 175.88	−28.63 ± 228.18	0.55	1497.58 ± 191.29	−3.83 ± 189.57	0.82	24.79 ± 61.33	0.69
Respiratory Quotient	0.82 ± 0.06	0.00 ± 0.07	0.76	0.82 ± 0.06	−0.01 ± 0.07	0.58	0.01 ± 0.02	0.54
Carbohydrate Oxidation (% REE kcal)	29.21 ± 10.37	−6.46 ± 6.67	<0.001	33.31 ± 14.86	3.83 ± 3.39	<0.001	6.63 ± 5.92	0.22
Fat Oxidation (% REE kcal)	56.68 ± 12.04	2.85 ± 3.66	<0.001	49.34 ± 16.03	−3.63 ± 3.57	<0.001	34.13 ± 13.73	0.27
Protein Oxidation (% REE kcal)	14.37 ± 4.64	5.97 ± 5.69	<0.001	17.81 ± 6.82	−1.41 ± 1.50	0.05	0.55 ± 1.94	0.77
Visceral Adipose Tissue Area (cm)	67.23 ± 41.92	−5.27 ± 13.05	0.06	57.56 ± 48.18	4.49 ± 13.08	0.10	7.78 ± 10.77	0.44
Subcutaneous Adipose Tissue Area (cm)	287.65 ± 110.56	−2.14 ± 42.11	0.81	244.27 ± 71.78	5.00 ± 30.89	0.44	7.14 ± 10.66	0.51
VAT:SAT (ratio)	0.23 ± 0.13	−0.02 ± 0.05	0.16	0.22 ± 0.16	−0.01 ± 0.08	0.30	0.02 ± 0.05	0.64

**Table 3:** Multivariable Regression Modeling of Treatment Effects of Tree Nuts Snacks on Metabolic Syndrome Risk in Young Adults.

Dependent Variable = Metabolic Syndrome Score						Dependent Variable = Triglycerides					
Source	Type III Sum of Squares	*df*	Mean Square	F	*p*-Value	Source	Type III Sum of Squares	*df*	Mean Square	F	*p*-Value
Corrected Model	67.139	4	16.785	60.955	<0.001	Corrected Model	210,000.721	4	52,500.180	21.072	<0.001
Intercept	0.017	1	0.017	0.063	0.80	Intercept	643.928	1	643.928	0.258	0.61
Met Sx (baseline)	52.613	1	52.613	191.066	<0.001	Triglycerides (baseline)	180,492.047	1	180,492.047	72.443	<0.001
Sex	2.345	1	2.345	8.517	0.005	Sex	8609.145	1	8609.145	3.455	0.07
Treatment = Tree Nuts	1.127	1	1.127	4.091	0.04	Treatment = Tree Nuts	9924.296	1	9924.296	3.983	0.04
Treatment * Sex	3.252	1	3.252	11.810	<0.001	Treatment * Sex	1978.462	1	1978.462	0.794	0.37
Error	21.754	79	0.275			Error	194,338.339	79	2491.517		
Total	167.000	84				Total	1,142,246.000	84			
Corrected Total	88.893	83				Corrected Total	404,339.06	83			
**Dependent Variable = Fasting Insulin Level**						**Dependent Variable = TG/HDL** **Ratio**					
**Source**	**Type III Sum of Squares**	***df***	**Mean Square**	**F**	***p*-value**	**Source**	**Type III Sum of Squares**	***df***	**Mean Square**	**F**	***p*-value**
Corrected Model	209.299	4	52.325	12.001	<0.001	Corrected Model	177.386	4	44.347	20.691	<0.001
Intercept	67.554	1	67.554	15.494	<0.001	Intercept	1.470	1	1.470	0.686	0.41
Insulin (baseline)	181.423	1	181.423	41.610	<0.001	TG/HDL ratio (baseline)	142.676	1	142.676	66.568	<0.001
Sex	0.006	1	0.006	0.001	0.97	Sex	5.193	1	5.193	2.423	0.12
Treatment = Tree Nuts	12.256	1	12.256	3.811	0.05	Treatment = Tree Nuts	9.138	1	9.138	4.264	0.04
Treatment * Sex	7.017	1	7.017	1.609	0.21	Treatment * Sex	2.989	1	2.989	1.395	0.24
Error	344.451	79	4.36			Error	167.178	79	2.143		
Total	1016.700	84				Total	714.978	84			
Corrected Total	553.750	83				Corrected Total	344.564	83			

## Data Availability

Because of the nature of the data collected for this study, requests to access the data set from qualified researchers trained in human subject confidentiality protocols may be sent to the corresponding author.

## Supplementary Materials

The following supporting information can be downloaded at: https://www.mdpi.com/article/10.3390/nu15245051/s1, Table S1: Changes in Dietary Energy and Nutrient Intakes.
