# Supplementary material for: Consumption of Tree Nuts as Snacks Reduces Metabolic Syndrome Risk in Young Adults: A Randomized Trial
Source: Nutrients. 2023 Dec 9;15(24):5051. doi: 10.3390/nu15245051 (PMC10745921; doi:10.3390/nu15245051)
Supplement: Supplementary file 1 [file nutrients-15-05051-s001.zip › nutrients-2733814-supplementary.pdf]

Supplemental Table. Changes in Dietary Energy and Nutrient Intakes in Males (n= 36)								
	Tree Nuts Group			CHO Group			Treatment Effect	
	**Baseline**	**Change**	***P-value***	**Baseline**	**Change**	***P-value***	**Mean Difference**	***P-value***
**Total Amount (g)**	3439.66 ± 1588.84	-377.45 ± 376.80	0.33	3256.45 ± 1367.81	213.18 ± 405.25	0.61	590.63 ± 565.01	0.29
**Energy (kcal)**	2491.77 ± 808.16	-169.49 ± 237.69	0.49	2473.52 ± 660.18	19.72 ± 153.35	0.90	189.20 ± 272.83	0.49
**Carbohydrate (% kcal)**	44.66 ± 9.90	-13.95 ± 3.67	0.002	46.70 ± 9.99	0.21 ± 2.49	0.93	14.16 ± 4.30	0.002
**Fat (% kcal)**	33.63 ± 4.12	13.03 ± 2.79	<0.001	32.76 ± 4.55	0.27 ± 1.83	0.88	12.75 ± 3.22	<0.001
**Protein (% kcal)**	18.91 ± 6.10	0.52 ± 2.29	0.82	18.94 ± 6.93	-0.01 ± 1.51	0.99	0.53 ± 2.65	0.84
**Animal protein (g)**	82.33 ± 52.67	0.01 ± 17.99	0.99	74.42 ± 40.11	1.63 ± 11.43	0.89	1.62 ± 20.52	0.94
**Vegetable protein (g)**	29.71 ± 13.50	-1.39 ± 3.83	0.72	34.69 ± 15.32	-1.25 ± 4.32	0.78	0.14 ± 5.78	0.98
**Saturated Fat (g)**	30.23 ± 18.95	0.21 ± 4.64	0.96	30.79 ± 13.79	-2.24 ± 4.08	0.59	2.45 ± 6.17	0.69
**MonoUnsaturated Fat (g)**	28.75 ± 12.29	28.77 ± 9.83	0.01	29.99 ± 10.36	-1.09 ± 3.52	0.76	29.68 ± 9.61	0.007
**PolyUnsaturated Fat (g)**	21.59 ± 6.70	5.30 ± 3.25	0.12	20.09 ± 5.69	4.11 ± 2.45	0.11	1.19 ± 3.99	0.77
**Saturated Fat (% kcal)**	10.48 ± 3.62	1.09 ± 3.78	0.27	10.86 ± 2.89	1.00 ± 1.02	0.34	0.09 ± 1.39	0.51
**MonoUnsaturated Fat (% kcal)**	10.23 ± 2.45	10.81 ± 2.06	<0.001	10.77 ± 2.60	0.08 ± 0.81	0.93	10.73 ± 2.05	<0.001
**PolyUnsaturated Fat (% kcal)**	7.90 ± 1.91	2.31 ± 0.88	0.02	7.29 ± 1.63	0.93 ± 0.63	0.16	1.37 ± 1.06	0.20
**Omega-3 Fats (g)**	2.11 ± 0.88	0.51 ± 0.48	0.31	2.31 ± 0.78	0.23 ± 0.30	0.46	0.28 ± 0.55	0.62
**Sucrose (g)**	43.76 ± 45.87	-17.75 ± 8.02	0.04	41.51 ± 32.31	-1.57 ± 7.76	0.84	16.18 ± 11.26	0.01
**Starch (g)**	128.54 ± 40.86	-49.84 ± 16.17	0.01	142.88 ± 40.66	0.10 ± 10.46	0.99	49.94 ± 18.58	0.01
**Total Fiber (g)**	21.19 ± 10.64	-0.62 ± 3.40	0.86	22.84 ± 6.64	1.05 ± 2.42	0.67	1.67 ± 4.06	0.68
**Soluble Fiber (g)**	6.71 ± 3.78	-1.26 ± 1.29	0.34	7.99 ± 3.46	-0.35 ± 0.95	0.72	0.91 ± 1.57	0.57
**Insoluble Fiber (g)**	13.89 ± 7.80	1.19 ± 2.49	0.64	14.65 ± 4.92	0.62 ± 1.71	0.72	0.57 ± 2.94	0.85
**Pectins (g)**	1.64 ± 1.22	2.01 ± 0.46	<0.001	2.22 ± 1.19	0.26 ± 0.35	0.47	1.75 ± 0.57	0.004
**Total Sugars (g)**	95.51 ± 68.96	-31.92 ± 14.71	0.04	101.03 ± 57.42	-6.32 ± 9.45	0.51	25.60 ± 16.86	0.04
**Added Sugars (g)**	61.60 ± 56.59	-24.85 ± 10.36	0.03	64.74 ± 54.65	-10.11 ± 11.80	0.40	14.74 ± 16.13	0.05
**Refined Grains (oz eq)**	6.98 ± 3.05	-2.51 ± 0.99	0.02	7.86 ± 3.89	-0.54 ± 1.05	0.61	1.97 ± 1.47	0.19
**Sodium (mg)**	4083.06 ± 1282.08	-392.14 ± 658.09	0.56	4574.82 ± 1866.17	18.86 ± 605.61	0.98	411.01 ± 897.26	0.65

Supplemental Table. Changes in Dietary Energy and Nutrient Intakes in Females (n= 48)								
	Tree Nuts Group			CHO Group			Treatment Effect	
	**Baseline**	**Change**	***P-value***	**Baseline**	**Change**	***P-value***	**Mean Difference**	***P-value***
**Total Amount (g)**	3316.08 ± 1569.99	-564.39 ± 347.81	0.12	3436.91 ± 1727.01	-310.07 ± 440.56	0.49	254.32 ± 558.72	0.65
**Energy (Kcal)**	1956.38 ± 559.59	49.51 ± 151.60	0.75	2205.34 ± 750.40	225.41 ± 173.60	0.21	175.89 ± 229.91	0.45
**Carbohydrate (% kcal)**	49.61 ± 9.52	-10.88 ± 2.06	<0.001	50.32 ± 6.03	-7.63 ± 1.84	<0.001	3.25 ± 2.77	0.25
**Fat (% kcal)**	34.56 ± 8.21	8.55 ± 1.86	<0.001	35.04 ± 5.73	3.69 ± 1.79	0.05	4.86 ± 2.58	0.05
**Protein (% kcal)**	15.36 ± 5.81	0.30 ± 1.30	0.82	14.11 ± 4.40	2.37 ± 1.07	0.03	2.07 ± 1.69	0.23
**Animal protein (g)**	45.47 ± 29.25	-2.37 ± 7.38	0.75	43.44 ± 19.82	21.19 ± 6.89	0.01	23.56 ± 10.12	0.01
**Vegetable protein (g)**	27.62 ± 10.65	4.47 ± 2.43	0.07	32.12 ± 14.13	-1.52 ± 3.38	0.66	5.99 ± 4.13	0.15
**Saturated fat (g)**	22.94 ± 10.50	-0.27 ± 2.44	0.91	28.28 ± 17.67	5.25 ± 4.72	0.28	5.52 ± 5.25	0.29
**MonoUnsaturated Fat (g)**	25.34 ± 10.56	16.22 ± 3.23	<0.001	27.77 ± 12.06	7.05 ± 14.85	0.03	9.17 ± 4.48	0.02
**PolyUnsaturated Fat (g)**	18.30 ± 10.67	7.55 ± 1.88	<0.001	22.08 ± 9.58	5.28 ± 3.47	0.14	2.27 ± 3.89	0.56
**Saturated Fat (% kcal)**	10.15 ± 3.50	-0.17 ± 3.66	0.83	10.91 ± 3.74	0.79 ± 5.31	0.48	0.95 ± 1.32	0.48

**MonoUnsaturated Fat (% kcal)**	11.35 ± 4.43	7.18 ± 1.27	<0.001	11.10 ± 2.43	1.43 ± 0.63	0.03	5.75 ± 1.43	<0.001
**PolyUnsaturated Fat (% kcal)**	7.83 ± 3.36	3.36 ± 0.78	<0.001	8.92 ± 2.81	1.02 ± 1.05	0.34	2.34 ± 1.30	0.05
**Omega-3 FA (g)**	2.28 ± 1.97	0.50 ± 0.29	0.10	2.64 ± 1.17	0.23 ± 0.43	0.60	0.27 ± 0.52	0.61
**Sucrose (g)**	39.37 ± 21.14	-5.94 ± 6.12	0.34	47.75 ± 34.47	-8.38 ± 8.05	0.31	2.44 ± 10.06	0.81
**Starch (g)**	118.68 ± 56.69	-26.43 ± 11.09	0.03	141.63 ± 59.38	-25.15 ± 16.66	0.15	1.28 ± 19.85	0.95
**Total Fiber (g)**	21.24 ± 7.84	0.21 ± 2.03	0.92	25.49 ± 9.87	-2.30 ± 2.55	0.38	2.51 ± 3.25	0.44
**Soluble Fiber (g)**	6.61 ± 2.80	-0.83 ± 0.65	0.21	7.34 ± 2.82	-0.74 ± 0.70	0.30	0.09 ± 0.96	0.93
**Insoluble Fiber (g)**	14.04 ± 5.98	1.29 ± 1.61	0.43	17.61 ± 8.65	-1.92 ± 2.06	0.36	3.20 ± 2.60	0.22
**Pectins (g)**	2.63 ± 1.39	1.03 ± 0.40	0.02	2.85 ± 1.90	-0.06 ± 0.55	0.91	1.09 ± 0.68	0.05
**Total Sugars (g)**	83.44 ± 32.37	-20.05 ± 7.10	0.01	93.32 ± 50.35	0.24 ± 12.56	0.99	20.29 ± 14.27	0.01
**Added Sugars (g)**	50.06 ± 26.54	-5.35 ± 6.67	0.43	59.50 ± 44.16	-4.63 ± 11.54	0.69	0.72 ± 13.19	0.95
**Refined Grains (oz eq)**	5.93 ± 3.70	-1.74 ± 0.77	0.03	6.24 ± 3.75	-0.76 ± 0.92	0.42	0.98 ± 1.20	0.42
**Sodium (mg)**	3254.98 ± 1225.87	-424.08 ± 285.20	0.05	4069.82 ± 2097.62	-168.63 ± 356.15	0.64	255.45 ± 454.31	0.58
